# CORN 2.0 - Condition Orientated Regulatory Networks 2.0

**DOI:** 10.1016/j.csbj.2025.04.003

**Published:** 2025-04-03

**Authors:** Ricky Wai Tak Leung, Xinying Zhang, Zhuobin Chen, Yuyun Liang, Simei Huang, Zixin Yang, Xueqing Zong, Xiaosen Jiang, Runming Lin, Wenbin Deng, Yaohua Hu, Jing Qin

**Affiliations:** a School of Pharmaceutical Sciences (Shenzhen), Shenzhen Campus of Sun Yat-sen University, Shenzhen, Guangdong 518107, China; b Division of Science, Engineering and Health Studies, College of Professional and Continuing Education, The Hong Kong Polytechnic University, Hong Kong SAR, China; c College of Life Sciences, University of Chinese Academy of Sciences, Beijing 100049, China; d BGI-Shenzhen, Shenzhen, Guangdong 518103, China; e School of Mathematical Sciences, Shenzhen University, Shenzhen, Guangdong 518060, China

**Keywords:** Personalized medicine, Induced pluripotency, Cell fate, Gene regulatory network, Natural products, Natural compounds, Chinese medicine, Herbal medicine, Tumor heterogeneity, Embryonic stem cells

## Abstract

Gene regulation is a fundamental process that allows organisms to adapt to their environment and increase complexity through the action of nucleic acid-binding proteins (NBPs), such as transcription factors (TFs), which regulate specific sets of genes under distinct conditions. These regulatory interactions form transcriptional regulatory networks (TRNs), which can be further broken down into transcriptional regulatory sub-networks (TRSNs) centered around individual TFs. TRSNs are more stable and practical for analysis, making them ideal for studying gene regulation under specific conditions. Condition-Oriented Regulatory Networks (CORN, https://qinlab.sysu.edu.cn/corn/home) is a comprehensive library of condition-based TRSNs, including those induced by natural compounds, small molecules, drug treatments, and gene perturbations. CORN 2.0 represents a significant update, associating 7540 specific conditions with 71934 TRSNs across 52 human cell lines, involving 542 transcription factors (TFs). Notably, CORN 2.0 includes 1550 natural compound-triggered TRSNs, providing a valuable resource for studying the pharmacological effects of natural products. This study demonstrates the utility of CORN in three key areas: personalized medicine, induced pluripotency transitions, and natural compound-associated pharmacology. By linking specific conditions to their corresponding TRSNs, CORN enables researchers to explore how gene regulatory networks are altered under various conditions, offering insights into disease mechanisms and potential therapeutic interventions.

## Highlights

• A 418 % and a 753 % increase in conditions and TRSNs curations respectively.
• CORN 2.0 associates 7540 different types of specific conditions to 71934 TRSNs in 52 human cell lines, involving 542 TFs.
• CORN aids personalized medicine by identifying optimal drugs for cancer cells, addressing tumoral heterogeneity.
• A list of chemicals can potentially be repurposed to aid fibroblast and primed-to-naïve pluripotency conversion.
• 1550 TRSNs are associated with 90 kinds of natural compounds/herb chemicals with chronic diseases therapeutic potential.

## Graphical Abstract

**Figure:**
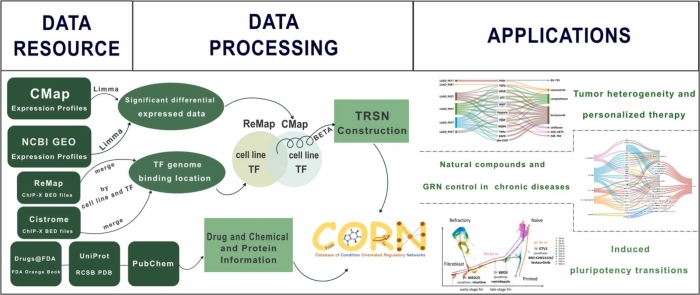

## 1 Introduction

### 1.1 Condition-Orientated Regulatory Networks

Gene regulation is essential for cellular adaptation and complexity, mediated by transcription factors (TFs) that control gene expression under specific conditions [42]. These interactions form transcriptional regulatory networks (TRNs), which can be further dissected into transcriptional regulatory sub-networks (TRSNs) centered around individual TFs. TRSNs are more stable and practical for analysis, making them ideal for studying gene regulation under specific conditions [33], [78]. CORN is an online platform of Condition Orientated Regulatory Networks [43] that provides a library of condition-based TRSNs, including those induced by natural compounds, small molecules, drug treatments, and gene perturbations. Compared to other similar databases, CORN is the first of its kind, offering unique functionalities and applications that are not replicated elsewhere including its extensive curation of natural compound-triggered TRSNs and its applicability in personalized medicine and drug discovery. For instance, databases like CMap [72] focus on transcriptomic data but do not construct regulatory networks, while RegNetwork [48] integrates pre-discovered linear regulatory relationships between genes without computationally constructing regulatory networks. Similarly, GRNdb [18] associates human and mouse transcription factors (TFs) with downstream genes by constructing transcriptional regulatory networks (TRNs) across various normal and pathological tissues using inferred TF binding data. However, neither GRNdb nor RegNetwork emphasizes drug/small molecule-induced or genetic perturbation-induced regulatory changes. While Kyoto Encyclopedia of Genes and Genomes (KEGG) [34] curates genomic, chemical, and signaling pathway information directly from literature and diverse data sources, it does not construct regulatory networks

KEGG pathway members may originate from different regulatory levels and are not necessarily co-expressed or directly interacting. In contrast, gene members within a CORN TRSN are co-regulated at the same regulatory level by the same TF under the same temporal conditions, ensuring direct regulatory relationships. In other words, drugs or genes curated in CORN exert direct control over gene members within their respective TRSNs, whereas members of the same KEGG pathway do not necessarily share direct regulatory interactions. Thus, CORN is a powerful tool for understanding gene regulation in response to chemical and genetic perturbations.

In this update, we report a 418 % increase in condition curation and a 753 % increase in TRSN curation, significantly expanding the scope and utility of CORN. The updated database now includes 1550 natural compound-triggered TRSNs, enabling researchers to explore the pharmacological effects of natural products.

CORN 2.0 also features an 11-fold increase in enriched TRSNs across a wide range of diseases, covering all 50 Disease Ontology (DO) categories, a 12-fold increase in associated KEGG pathways, and a 33-fold increase in related TFs. These enhancements make CORN a powerful tool for studying gene regulatory networks in pathological and pharmaceutical research.

This study demonstrates the utility of CORN in three key areas: personalized medicine, induced pluripotency transitions, and natural compound-associated pharmacology. By linking specific conditions to their corresponding TRSNs, CORN enables researchers to explore how gene regulatory networks are altered under various conditions, offering insights into disease mechanisms and potential therapeutic interventions.

### 1.2 Tumor heterogeneity and personalized therapy

Malignant tumors are increasingly prevalent and deadly, posing a significant global health challenge [73]. Despite advancements in anti-cancer therapies and chemotherapy regimens that have improved cure rates, tumor heterogeneity remains a major obstacle to effective cancer diagnosis and treatment. This heterogeneity manifests as cellular and molecular differences among molecular subtypes and even among patients with the same cancer type [67]. Such variability leads to differential drug responses, complicating treatment strategies. However, the discovery of unique drug-controlled transcriptional networks offers a promising avenue to counteract cancer heterogeneity, underscoring the importance of personalized therapy [52]. CORN’s extensive drug-network association data in cancer cells provides a valuable resource for researchers and clinicians to develop tailored treatment recommendations.

### 1.3 Induced pluripotency transitions

Over the past decade, stem cell research has made remarkable strides. Human induced pluripotent stem cells (iPSCs), generated by genetically reprogramming terminally differentiated somatic cells into embryonic-like cells using pluripotency genes, have shown immense potential in regenerative medicine [11], disease modeling [83], and human developmental biology [12]. Recent advances have demonstrated that small molecules can safely replace traditional reprogramming factors to induce pluripotency. For instance, in 2013, a team identified seven small molecules from tens of thousands of candidates to replace all transcription factors required for converting mouse fibroblasts into iPSCs [32]. A similar breakthrough was achieved in 2022 with human cells [25]. Moreover, in 2023, researchers successfully utilized chemically induced reprogramming to reverse cellular aging [87]. Chemical reprogramming offers advantages such as reduced time, improved efficiency, simplified processes, and lower costs. However, challenges remain in applying this approach to clinically valuable cell types. To address these limitations, we propose that dissecting gene regulatory networks in these cells could enable scientists to overcome current barriers and explore novel methods for manipulating cell fate [59]. CORN provides essential resources for studying drug-induced cell state transitions through gene regulation, enhancing our understanding of the fundamental mechanisms underlying chemical reprogramming.

### 1.4 Natural compounds

Natural products (NPs), derived from plants, animals, and microorganisms, are a rich source of physiologically active compounds [2]. Historically, natural compounds like aspirin have served as foundational medications. The intricate molecular frameworks of natural products offer medicinal chemists a diverse array of chemotypes for developing chemical probes and drugs [14], [16], [41], [5]. Notably, over 30 % of FDA-approved drugs between 1981 and 2019 were natural products or their derivatives, highlighting their critical role in areas such as anti-infectives and anti-tumor therapies [35], [54]. However, the lack of target interaction data for many natural products has hindered their application in drug discovery. Computational approaches now enable system-level compound-target interaction analysis on a large scale, offering a cost-effective alternative to traditional wet lab experiments. CORN’s comprehensive systems-based platform bridges natural compounds with gene regulatory networks, facilitating the understanding and repurposing of these molecules for therapeutic applications.

## 2 Results and Discussion

### 2.1 Platform update

In this update, we report a 418 % increase in condition curation and a 753 % increase in TRSN curation. CORN 2.0 now features 1550 natural compound-triggered TRSNs. In total, CORN 2.0 associates 7540 specific conditions—including natural compounds, small molecules, drug treatments, and gene perturbations—with 71934 TRSNs across 52 human cell lines, involving 542 transcription factors (TFs). A summary is provided in Table S1.

**Fig. 1:**
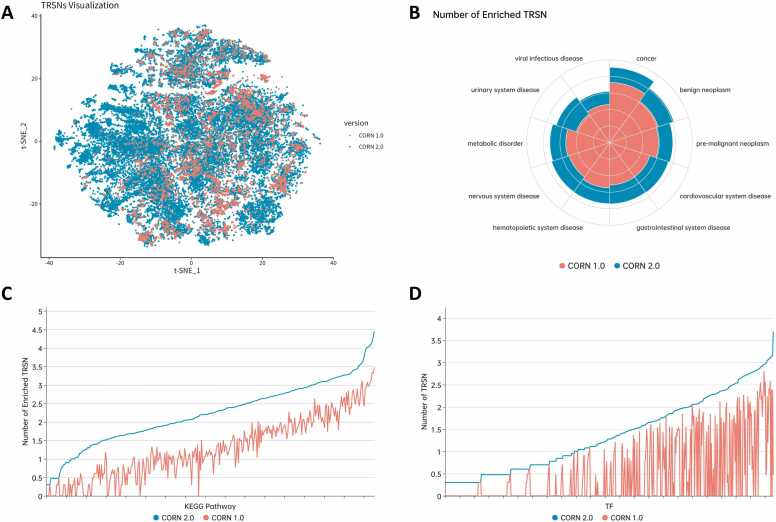
Comparisons between CORN 2.0 (cyan) and CORN 1.0 (red). (A) TRSNs curated in CORN visualized by t-distributed stochastic neighbor embedding (t-SNE). (B) Number of TRSNs associated with respective Disease Ontology (DO) disease categories in log10 scale. (C) Number of TRSNs enrichment with respective Kyoto Encyclopedia of Genes and Genomes (KEGG) pathway in log10 scale. (D) Number of TRSNs associated with respective TF in log10 scale.

CORN 2.0 significantly expands its coverage of cellular states, with an 11-fold increase in enriched TRSNs (Fig. 1A) across a wide range of diseases, encompassing all 50 Disease Ontology (DO) categories (Fig. 1B and Figure S1). It also features a 12-fold increase in associated KEGG pathways (Fig. 1C) and a 33-fold increase in related TFs (Fig. 1D). To enhance user experience, we have updated the web interface, introducing Browse and Application modules. The Browse module allows users to explore data using condition categories, tissue locations, and cell line filters, while the condition matching module provides insights into the uses of CORN in drug mechanism research and discovery.

### 2.2 Exploration of tumor heterogeneity and personalized medicine

Tumor heterogeneity arises from biochemical and genetic alterations during tumor development, leading to variations in growth rates, invasive potential, and treatment sensitivity among cancer cells [50]. Two primary types of tumor heterogeneity exist: intratumoral heterogeneity, which refers to differences among cancer cells within the same patient, and intertumoral heterogeneity, which encompasses differences among patients with the same histological subtype. While bulk RNA sequencing has been used to study tumor heterogeneity, it measures average signals from mixed cell populations, masking individual cell variations. Single-cell RNA sequencing (scRNA-seq) offers a superior approach by capturing transcriptional variations at the single-cell level, enabling a more detailed analysis of tumor tissue composition and molecular characteristics [33], [58].

**Fig. 2:**
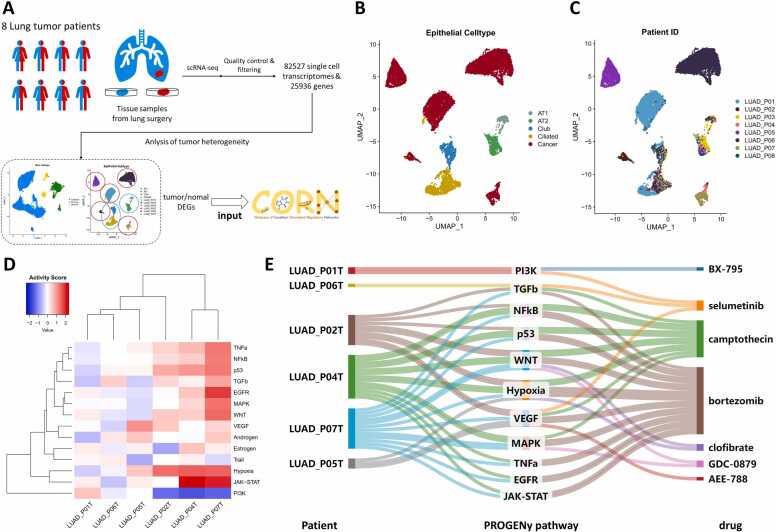
The tumor heterogeneity and personalized medicine of lung adenocarcinoma. (A) Schematic representation of the workflow, 8 pairs of normal (blue) and tumor (red) tissue samples were extracted from 8 patients. UMAPs based on the epithelial cells of all single-cell transcriptomes, color-coded by (B) cell type and (C) patient, where AT1 stands for Type Ⅰ alveolar epithelial cells; AT2 stands for Type Ⅱ alveolar epithelial cells; and LUAD_P01 stands for lung adenocarcinoma patient 01. (D) Heatmap of PROGENy pathway activity in each tumor subtype from different patient sources. (E) The connections between tumor subtypes from different patient sources, PROGENy pathways, and matched drugs. See main text for details.

To investigate lung adenocarcinoma heterogeneity, we analyzed a dataset of eight paired clinical samples from the Code Ocean database using scRNA-seq. Uniform Manifold Approximation and Projection (UMAP) analysis revealed 18 distinct cell clusters (Figure S2A), which were annotated as epithelial, immune, or stromal cells based on tumor microenvironment components and marker gene expression (Figure S2B). Focusing on epithelial cells, we identified five subtypes: Type I alveolar epithelial cells (AT1), Type II alveolar epithelial cells (AT2), club cells, ciliated cells, and cancer cells, using CNV scores and marker genes (Figure S2D). Normal epithelial cells from healthy lung samples served as references, confirming the accuracy of subtype annotations (Figure S2E). Notably, tumor cells exhibited patient-specific clustering, highlighting intertumoral heterogeneity (Fig. 2C). Using the R package PROGENy, we assessed the activity of 14 oncogenic signaling pathways across tumor cell subtypes (Fig. 2D)[66] (higher scores indicate greater activity of the respective pathways in the tumor subtype). Significant variations were observed in the activity of EGFR, JAK/STAT, PI3K, and Hypoxia pathways.

To demonstrate CORN’s utility in analyzing tumor heterogeneity and guiding personalized therapy, we submitted differentially expressed genes (DEGs) between tumor cells and normal epithelial cells to the TRSN matching tool. TRSNs with positive scores identified conditions or drugs that could correct disease-related gene expression changes [43]. Among the results, the NFIL3/PrPc axis, which regulates lung cancer invasiveness and metastasis via JNK signaling [46], was identified in three patients. CORN suggested selumetinib, an MEK1/2 inhibitor, as a potential regulator of NFIL3 and its downstream TRSN [30].

Patients LUAD_P04 and LUAD_P07 exhibited similar oncogenic pathway activities and were matched with bortezomib and GMPS gene perturbation (Table S2). Bortezomib, a proteasome inhibitor, has demonstrated significant antitumor activity [60], while GMPS, a key enzyme in guanosine monophosphate biosynthesis, is a promising therapeutic target. These findings illustrate CORN’s ability to align treatment recommendations with tumor heterogeneity, emphasizing the need for patient-tailored therapies [6].

### 2.3 Insights into stem cell manipulations

Pluripotent stem cells (PSCs), which possess the remarkable ability to differentiate into cells from all three embryonic germ layers, hold immense promise for regenerative medicine and developmental biology [29]. These cells exist in two distinct states: naïve and primed. Naïve PSCs, characterized by their unrestricted differentiation capacity, offer greater biomedical potential compared to primed PSCs, which are developmentally mature and exhibit lineage bias during differentiation, thereby limiting their applications [23], [55]. Conventional human embryonic stem cells (hESCs) derived from pre-implantation embryos are typically considered primed [81]. Consequently, identifying extrinsic cues capable of reprogramming somatic cells into pluripotent stem cells and priming them into the naïve state represents a significant area of research with profound biomedical implications.

**Fig. 3:**
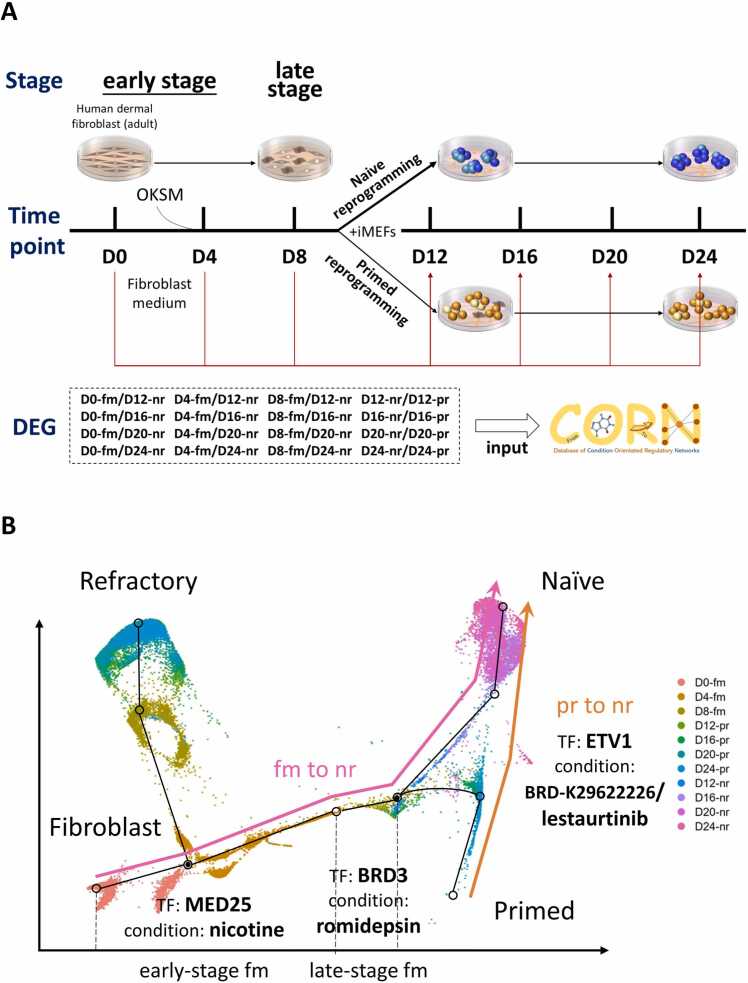
The experimental design and roadmap of stem cell reprogramming. (A) The experimental design for screening stem cell reprogramming chemical factors, modified from [47]. [Abbreviations: D, day; OKSM, OCT4, KLF4, SOX2 and MYC; iMEFs, irradiated mouse embryonic fibroblasts; fm, fibroblast medium; nr, naïve reprogramming; pr, primed reprogramming.] (B) Roadmap of human stem cell conversions with respective matched TFs and conditions. See main text for details.

In a clinically relevant study [47], cultured human dermal fibroblasts in media designed for primed (KSR with FGF2) and naïve (t2iLGoY) reprogramming, as illustrated in Fig. 3A. The researchers collected intermediates at days 0, 4, 8, 12, 16, 20, and 24, followed by single-nucleus RNA sequencing (snRNA-seq). The processed data from this study is available in the NCBI GEO database under accession number GSE147564. To explore whether small molecules, drugs, or specific conditions could influence stem cell induction and conversion, transcriptomic changes in the form of differentially expressed genes (DEGs) recorded during iPSC induction and conversion were analyzed using the TRSN matching tool in CORN. The results, presented in Table S3, indicate that higher matching scores reflect a closer resemblance between the TRSN and the differential expression profile. Fig. 3B outlines the roadmap for fibroblast-to-pluripotent stem cell conversion and primed-to-naïve transition, highlighting CORN’s recommended chemical treatments to facilitate these processes.

The identification of external cues capable of reprogramming somatic cells into pluripotent stem cells and inducing the naïve state holds significant biological and therapeutic potential. To investigate this, transcriptomic changes in the form of DEGs during iPSC induction and conversion were analyzed using the TRSN matching tool in CORN. Early-stage fibroblast-to-naïve state expression changes (starting from day 0 or day 4) were evaluated, with positive scores indicating that the input DEGs were counter-regulatory to the matched TRSNs. Notably, Mediator Complex Subunit 25 (MED25) appeared eight times in early-stage fibroblast-to-naïve state changes with high scores (Table S3), suggesting its critical role in this transition. MED25, an epigenetic regulator of H3K27 status, can dissociate polycomb repressive complexes [17] and has been identified as a master regulator of differentiation processes [64]. According to our platform, MED25-associated TRSNs can be modulated through a 24-hour nicotine treatment (Table S3).

Similarly, High Mobility Group Box 2 (HMGB2), a senescence regulator [26] appeared six times in early-stage changes with high scores (Table S3). HMGB2 is known to regulate differentiation, stemness [53], genomic organization, and CTCF clustering [90], as well as interact with OCT4 to regulate pluripotency [8]. Our platform indicates that HMGB2-associated TRSNs can be controlled using midostaurin, an antineoplastic agent [71]. Specificity Protein 1 (SP1), which appeared five times in early-stage changes with high scores (Table S3), has been shown to bind to the NANOG proximal promoter and activate its transcription [84]. NANOG is a master regulator of self-renewal and pluripotency [63]. SP1 and its downstream TRSNs can be manipulated using a 24-hour treatment of BRD-K49669601. Additionally, Negative Elongation Factor E (NELFE), a regulator of RNA Pol II promoter-proximal pausing [10], appeared three times in early-stage changes with high scores (Table S3). NELFE regulates MYC and its targets [13], with MYC being a key factor in pluripotency maintenance [9]. A six-hour treatment of BRD-K96977520 can modulate NELFE-associated TRSNs. In summary, treatments with nicotine, midostaurin, BRD-K49669601, and BRD-K96977520 show potential for facilitating early fibroblast-to-naïve stem cell conversion without genetic alterations.

For late-stage fibroblast-to-naïve state expression changes (starting from day 8 or later), Bromodomain Containing 3 (BRD3) appeared three times with high scores (Table S3). BRD3 promotes pluripotent molecular signatures, nuclear reprogramming, and mitosis regulation [68] while attenuating iPSC reprogramming stress [31]. A 24-hour treatment of romidepsin, a natural product, can control BRD3-associated TRSNs [20], [77]. Additionally, FOS Like 2, AP-1 Transcription Factor Subunit (FOSL2), showed high scores in late-stage changes. FOSL2 is involved in the epigenetic and transcriptional regulation of pluripotent stem cell differentiation, and its TRSNs can be modulated using the anti-cancer proteasome inhibitor MLN-9708 [21]. In conclusion, romidepsin and MLN-9708 treatments hold potential for facilitating late-stage fibroblast-to-naïve stem cell conversion.

Given the greater biomedical potential of naïve pluripotent cells due to their unrestricted differentiation capacity and lack of epigenetic limitations, prime-to-naïve pluripotency conversion is highly desirable. When naïve/primed state expression changes were analyzed using the condition matching tool, negative scores indicated alignment between the regulatory direction of the input DEGs and the reference TRSNs. FOSL2-associated TRSNs showed high negative scores in naïve/primed changes (Table S3), suggesting its role in facilitating prime-to-naïve conversion. ETS Variant Transcription Factor 1 (ETV1), a member of the ETS TF family, appeared consistently in naïve/primed changes (Table S3). ETV1 regulates cellular growth, proliferation, and differentiation, and its manipulation via BRD-K29622226 or lestaurtinib shows promise. Yes-Associated Protein 1 (YAP1), which plays a critical role in stem cell self-renewal and differentiation [45], also showed a high negative score in naïve/primed changes (Table S3). YAP1-associated TRSNs can be modulated using camptothecin, a natural product with topoisomerase I inhibitor properties [44]. In summary, treatments with MLN-9708, BRD-K29622226, lestaurtinib, and camptothecin have the potential for repurposing in prime-to-naïve pluripotency conversion.

In conclusion, CORN demonstrates significant potential for screening chemically reprogramming compounds to facilitate various cell state conversions.

### 2.4 Natural compounds and gene regulatory network control in chronic diseases

**Fig. 4:**
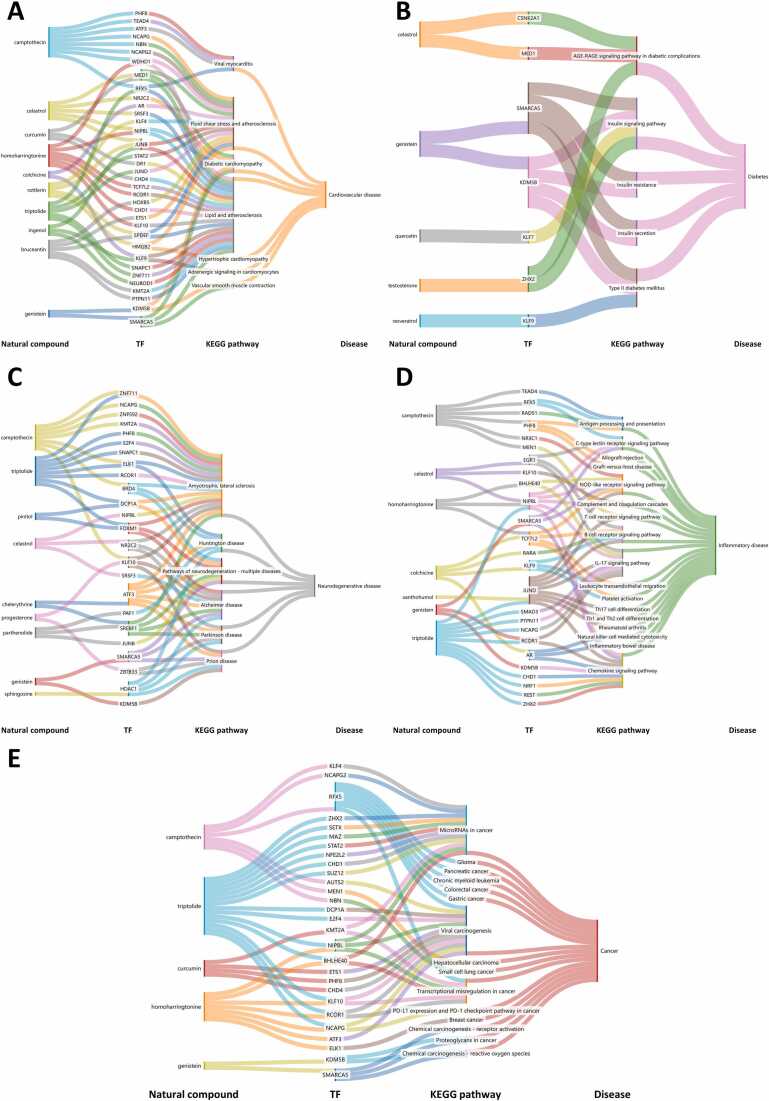
The connections between natural compounds, drug-targeted TFs, annotated KEGG pathways involved and pathological conditions of (A) cardiovascular diseases, (B) diabetes, (C) neurological diseases, (D) inflammatory diseases and (E) cancers. Specific details is provided in Table S4.

**Fig. 5:**
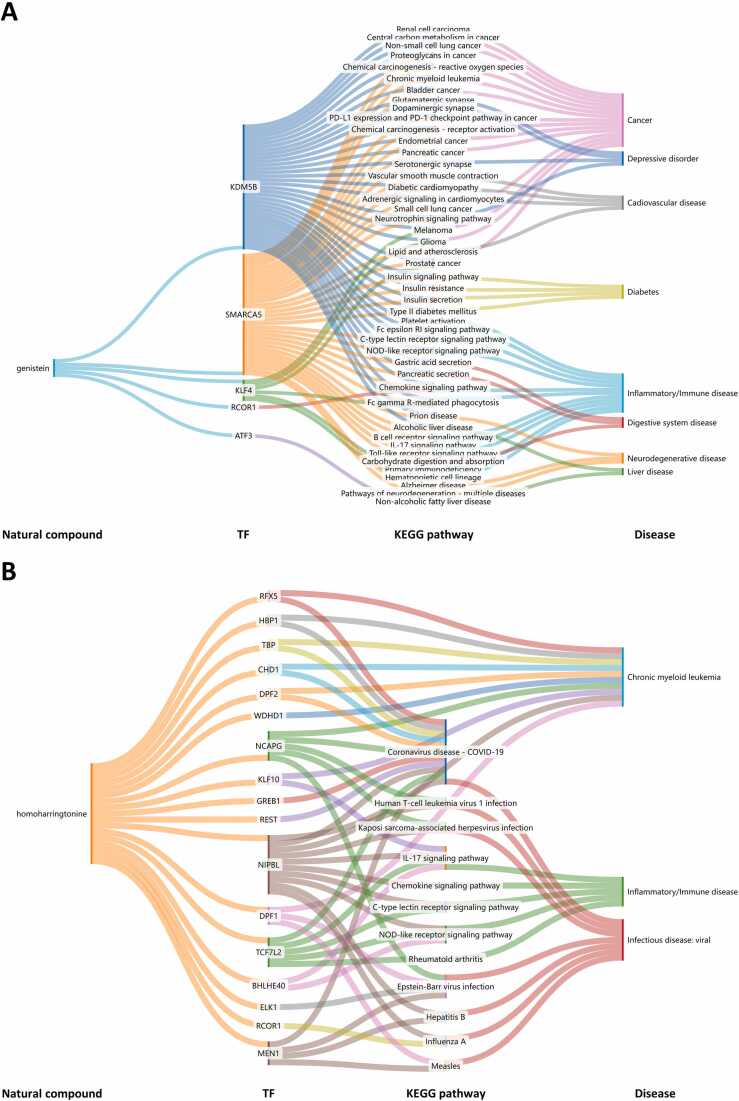
The connections between (A) genistein, TFs and different pathological conditions. (B) homoharringtonine, TFs, annotated KEGG pathways involved, and different pathological conditions. Specific details is provided in Table S5.

The global use of herbal medicine is on the rise [82] yet the mechanisms by which many herbal compounds and natural substances exert their effects on human cells remain poorly understood. To elucidate how natural chemicals influence gene regulatory networks for drug discovery and repositioning, we utilized our platform to perform Disease Ontology and KEGG enrichment studies on regulated genes within 1550 TRSNs associated with 90 natural compounds across 23 human cell lines. This analysis identified CORN disease-rectifying mechanisms linked to natural compounds, which are implicated in addressing gene network disorders such as cardiovascular disease, diabetes, neurological disorders, inflammatory diseases, and cancer (Fig. 4). Approximately 40 natural compounds exhibit anti-cancer potential, with five showing significant enrichment in cancer signaling pathways, as illustrated in a Sankey diagram (Fig. 4E). Notably, compounds like genistein and homoharringtonine demonstrate therapeutic potential for multiple diseases (Fig. 5).

Genistein is a multi-mechanistic phytochemical [69], exhibits protective effects against inflammatory, neurological, cardiovascular, diabetic, climacteric, and cancerous conditions [1], [15], [22], [24], [3], [40], [49]. Our study revealed that genistein-associated TRSN gene sets are enriched in malignancy-related biological processes, including PD-L1 expression, checkpoint pathways, chemical carcinogenesis, and central carbon metabolism. Many genes within these TRSNs belong to the MAPK signaling pathway, a key cascade in oncogenesis. Additionally, the chromatin remodeling factor SMARCA5, implicated in various pathogenic processes beyond neurological disorders, was identified as a significant player [51], [70].

Homoharringtonine, derived from the southern Chinese plant Cephalotaxus mannii, has been used since the 1970s to treat acute myeloid leukemia (AML) and chronic myeloid leukemia (CML) [19], [80], [86]. Fig. 5B illustrates how the transcription factor NIPBL mediates a complex gene regulatory network linking homoharringtonine to CML treatment. The CORN database further suggests that homoharringtonine may have potential applications in treating inflammatory and infectious diseases, warranting further investigation.

### 2.5 Future development of CORN

CORN 2.0 represents a significant advancement in the study of gene regulatory networks and their applications in personalized medicine, cell fate manipulation, and natural compound pharmacology. However, the platform is poised for further development to address emerging challenges and expand its utility. One of the primary goals for CORN is to expand its database to include a broader range of conditions, cell types, and disease states. This will involve curating additional transcriptional regulatory sub-networks (TRSNs) triggered by novel small molecules, natural compounds, and genetic perturbations. Furthermore, incorporating single-cell RNA sequencing (scRNA-seq) data from diverse tissues and disease models will enhance the platform’s ability to capture cellular heterogeneity and provide more precise insights into disease mechanisms. To improve user experience and analytical capabilities, CORN plan to introduce new functionalities, such as advanced visualization tools for exploring TRSNs, interactive pathway mapping, and machine learning-based prediction modules. These tools will enable researchers to identify novel drug-target interactions, predict therapeutic outcomes, and uncover hidden regulatory mechanisms. Additionally, integrating multi-omics data (e.g., proteomics, epigenomics, and metabolomics) will provide a more holistic understanding of gene regulation and its role in disease. A user-friendly interface with customizable workflows will further democratize access to CORN 2.0, making it a valuable resource for both computational biologists and experimental researchers. Despite its potential, CORN faces several challenges that must be addressed to fully realize its capabilities. One major limitation is the reliance on existing datasets, which may lack diversity in terms of cell types, conditions, and disease states. Expanding the database will require significant computational resources and collaboration with data providers. Additionally, the integration of multi-omics data presents technical challenges, including data standardization and the development of robust analytical pipelines. Finally, ensuring the accuracy and reliability of predictions generated by CORN will require continuous validation using experimental data and user feedback. In summary, the future development of CORN holds great promise for advancing our understanding of gene regulatory networks and their applications in biomedicine. By expanding the database, implementing new functionalities, and exploring novel applications, CORN has the potential to drive innovation in drug discovery, personalized medicine, and regenerative therapies, ultimately improving patient outcomes and advancing human health.

## 3 Conclusion

CORN 2.0 represents a significant advancement in the study of gene regulatory networks, with expanded condition and TRSN curation. The database’s applications in personalized medicine, stem cell research, and natural compound pharmacology demonstrate its broad utility in biomedical research. By linking specific conditions to their corresponding TRSNs, CORN enables researchers to explore how gene regulatory networks are altered under various conditions, offering insights into disease mechanisms and potential therapeutic interventions.

Future developments will focus on expanding the database, improving user experience, and exploring new applications. In short, CORN 2.0 is a powerful tool for studying gene regulatory networks in pathological and pharmaceutical research, with the potential to drive innovation in drug discovery and personalized medicine.

## 4 Methods

### 4.1 Data retrieval

A total of 11349 BED files associated with human TF ChIP-X data were downloaded from Cistrome Data Browser [89] and ReMap 2022 [28] in 2022 September. Transcriptomic data originated from 33889 interference methods (31661 small molecule compounds, 329 biological products, 525 shRNA, etc.) targeting 12328 target genes in human cell lines were downloaded from the NCBI GEO [4] and the Expanded CMap LINCS Resource released in December 2020 [72]. Chemical and physical information of different drugs and molecules were downloaded from PubChem [36]. Information of drugs in clinical phases were retrieved from Drugs@FDA and FDA Orange Book. Structural information of drug target proteins and the TFs were fetched from UniProt [76] and the Research Collaboratory for Structural Bioinformatics Protein Data Bank (RCSB PDB) [7]. Biological pathways associated with the drug target gene and the TF were retrieved from KEGG [34]. Transcriptomic data for drug screening was downloaded from NCBI GEO [4], Code Ocean and OMIX database.

### 4.2 Data processing

**Fig. 6:**
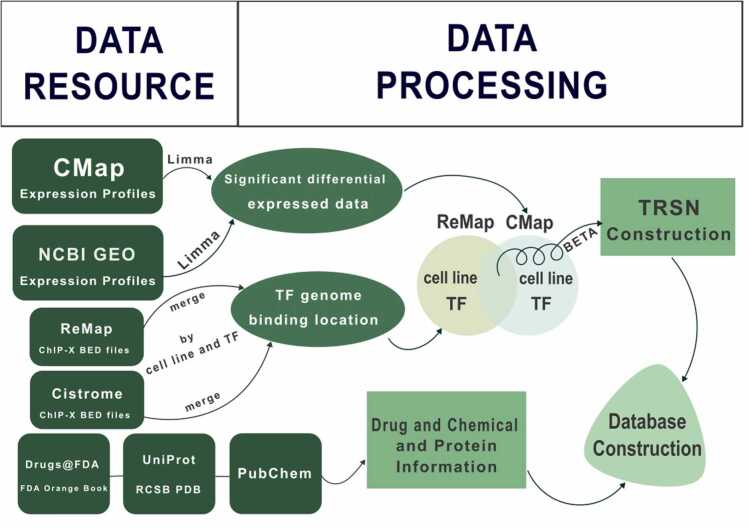
The construction process of CORN 2.0. Data sources, processing steps, TRSN construction, and external tools used at each stage are summarized in here.

ReMap 2022 integrates all non-redundant peaks of human TFs into a single file. The last column of the file provides annotation information indicating which TF produced the peak in which cell line. According to this annotation information, we separated the peak information into separate files of different cell lines and TFs. As for data from Cistrome, we merged BED files according to cell line and TF. It was found that there is partial overlap between ChIP-X data from two different sources. Since the peaks in ReMap 2022 have been processed to at least 25 % bi-directional overlap and the data source is relatively recent, we considered the data from ReMap 2022 to be more reliable. Therefore, for ChIP-X data exists in both databases, we adopted the data from ReMap 2022. After matching the transcriptomic data and ChIP-X data, 80 human cell lines were found to be included in both types of data. Thus, data originated from these cell lines were utilized for further data processing. ChIP-X data from the same cell line were merged and classified under the same BED file, while overlapping regions in a BED file were merged. Then, the merged regions in BED files were sorted according to their positions at chromosomes they originated from. An R package Limma [61] was used to conduct differential expression analyses on the transcriptomic data from NCBI GEO and Cmap to obtain Log2-transformed expression changes and adjusted *p*-values for each gene under different conditional treatment (using functions of lmfit and eBayes). Genes were considered differentially expressed with the absolute Log2 fold change value greater than 0.5 and the adjusted *p*-value less than 0.01. (Fig. 6)

### 4.3 TRSN constructions

Significant differential expressed data resulting from Limma (absolute value of Log2 fold change > 0.5 and adjusted *p*-value < 0.01) were selected to perform the following processes. ChIP-X and transcriptomic data from the same cell line were then paired and after target analysis by integration of both data with BETA [79]. Log2-transformed expression changes and adjusted *p*-values of all genes reported by Limma were parsed into BETA compatible format. Condition-specific TRSNs were constructed using BETA 1.0.7 with each pair of ChIP-X and transcriptome data, linking each differential expressed TF to their respective 200 most significant up-regulated and 200 most down-regulated genes under each specific condition into two TRSNs (One set of up-regulated genes containing TRSN, another set of TRSN with down-regulated genes). In this process, the parameters of BETA were set as basic, -k LIM, -da 200 utilizing genome information from *Homo sapiens* (human) genome assembly GRCh38 (hg38). (Fig. 6)

### 4.4 Database update

The web is constructed based on the powerful PHP framework CodeIgniter, which provides an Application Programming Interface (API) to connect the web to MySQL database. JavaScript libraries including JQuery, jQuery labelauty and Apache ECharts were used for data visualization. The overall matching matrix, which comprises the expression information of all TRSNs in CORN 2.0, has been divided into multiple submatrices for different sets of conditions and different sets of diseases.

### 4.5 Disease Ontology and KEGG enrichment analyses

t-SNE [37] was employed to show the differences between version 2.0 and version 1.0. Disease Ontology (DO) and KEGG enrichment were conducted to identify the diseases and pathways covered by each TRSN in version 2.0 and version 1.0. DO analysis was performed using the R package DOSE [88]. Then, the R package clusterProfiler [85] was used for KEGG enrichment analysis. Next, the enrichment of disease categories, specific diseases, pathways, and central TFs for each TRSN was compiled, analyzed and then visualized in log10 scale.

KEGG enrichment analyses were also conducted to search disease-related pathways natural compounds involved. KEGG analyses were performed using the R package clusterProfiler [85]. Then, Sankey diagrams were created in the SankeyMATIC web-based tool to show the connections between natural compounds, annotated drug target genes in our platform, annotated KEGG pathways involved and pathological conditions.

### 4.6 Cell state conversion key factors screening

The processed single-cell transcriptome data downloaded from NCBI GEO was read by the readRDS function [47]. Next, differential expression analyses were performed between cells sampled at different time points and between different cell states by the FindMarkers function with the following parameters applied: fraction of expressing cells ≥ 0.1, log fold change between cell populations ≥ 0.25. Finally, the identified DEGs and their Log2 fold change values were inputted into the matching tool of CORN. The magnitude of the resulting score reflects the degree of resemblance between the TRSN and the differential expression profile. A negative score indicates that the regulatory direction of the inputted differentially expressed genes (DEGs) aligns with the regulatory direction of the reference TRSN. On the other hand, a positive score indicates that the inputted DEGs exhibit a counter-regulatory direction relative to the matched TRSN.

### 4.7 The exploration of tumor heterogeneity and personalized medicine

Single-cell transcriptome datasets of lung adenocarcinoma which includes normal/adjacent and tumor samples, were collected and analyzed using the same set of pipelines as follow. The R package Seurat [62] was used to remove low-quality cells and genes with the following filtering criteria: cells with 500–9000 gene, 1000 - 100000 UMI, fraction of mitochondrial genes < 20 %, and log_10_GenesPerUMI > 0.8. The filtered genetic barcode matrix was standardized using the NormalizedData function in the R package Seurat with default parameters. Next, the FindVariableFeatures function in the Seurat package was deployed to identify the top 2000 most variable genes for PCA dimensionality reduction. The R package Harmony [38] is utilized for data integration, eliminating batch effects between different patients and different sequencing batches. Then, the FindNeighbors and the FindClusters function in the Seurat package were applied to perform graph-based clustering on the data after the dimension reduction with PCA. Nonlinear dimensionality reduction was applied by using the RunUMAP function and the results on two-dimensional UMAP was visualized by using the Dimplot function. The cell type annotations were based on the composition of the tumor microenvironment and the cells were classified into three major cell types: epithelial cells, immune cells and stromal cells based on classical marker genes: epithelial cells (EPCAM, SFN, KRT19), immune cells (PTPRC, CD3E, CD79A), and stromal cells (PECAM1, CD34, VWF, ACTA2, FAP, THY1) [39], [65], [74], [75]. For epithelial cell subtype annotations, dimensional reduction and re-clustering were performed. Subsequently, the epithelial cells were reannotated based on the marker genes specific to epithelial cell subtypes in lung from a published literature [27]:AT1 (AGER, PDPN), AT2 (ABCA3, SFTPC), club cells (SCGB1A1, MUC5B), and ciliated cells (FOXJ1, TMEM190). Additionally, the copy number variations (CNV) status of each epithelial cell was estimated by the InferCNV R package [56], which was the basis of distinguishing between normal cells and tumor cells. The CNV score of each cell was calculated by following the approach in Peng et al. Peng [57]. In order to further assess the differences in oncogenic signaling pathways among different tumor cell subtypes, we utilized the R package PROGENy [66] to evaluate the activity of cell subtypes on 14 classical oncogenic pathways (Androgen, Estrogen, Hypoxia, EGFR, STAT, MAPK, NFkB, PI3K, p53, TNF-α, TGF-β, Trail, VEGF, WNT). Then, a heatmap was generated to visualize the activity of oncogenic pathways across different tumor subtypes. DEGs between patient-specific tumor epithelial cells and all normal epithelial cells were computed using the FindMarkers function with the following parameters: fraction of expressing cells ≥ 0.1, log fold change between cell populations ≥ 0.25. The identified DEGs and their Log_2_ fold change values were inputted into the matching tool of CORN [43].

## Author contributions

RWTL interpreted the data, drafted and revised the manuscript. XZ and ZC conducted three application studies and drafted the manuscript. YL, SH, ZY and XZ collected multiple omics data and updated the database. XJ and RL updated the online platform. WD, YH and JQ contributed to the study design and manuscript revision.

## Disclosure Declaration

The authors declare that the research was conducted in the absence of any commercial or financial relationships that could be construed as a potential conflict of interest.

## Funding

This research was funded by the 10.13039/501100001809National Natural Science Foundation of China (32170655, 12222112, 12426311), the 10.13039/501100003453Natural Science Foundation of Guangdong Province (2024A1515011210), Project of Educational Commission of Guangdong Province of China (2023ZDZX1017), Shenzhen Science and Technology Program (RCJC20221008092753082, RCYX20231211090222026, JCYJ20241202124209011 and project No. 202206193000001, 20220817122906001), Research Team Cultivation Program of Shenzhen University (2023QNT011).

## CRediT authorship contribution statement

**Ricky Wai Tak Leung:** Conceptualization, Writing – original draft, Writing - Review & Editing, Project administration. **Xinying Zhang:** Writing – original draft, Writing - Review & Editing, Visualization. **Zhuobin Chen:** Data Curation, Writing - Review & Editing, Investigation, Visualization. **Yuyun Liang:** Writing - Review & Editing, Supervision. **Simei Huang:** Formal analysis, Investigation. **Zixin Yang:** Methodology, Validation. **Xueqing Zong:** Methodology, Validation. **Xiaosen Jiang:** Software. **Runming Lin:** Software. **Wenbin Deng:** Writing - Review & Editing, Supervision. **Yaohua Hu:** Conceptualization, Writing - Review & Editing, Supervision, Funding acquisition. **Jing Qin:** Conceptualization, Writing - Review & Editing, Supervision, Project administration, Funding acquisition.

## Declaration of Competing Interest

The authors declare that they have no known competing financial interests or personal relationships that could have appeared to influence the work reported in this paper.

## Data Availability

The data and codes underlying this article are available at https://qinlab.sysu.edu.cn/corn/home
