# Supplementary material for: CORN 2.0 - Condition Orientated Regulatory Networks 2.0
Source: Comput Struct Biotechnol J. 2025 Apr 3;27:1518–28. doi: 10.1016/j.csbj.2025.04.003 (PMC12017979; doi:10.1016/j.csbj.2025.04.003)
Supplement: Supplementary file 1 — Supplementary material [file mmc1.pdf]

Supplementary Materials

CORN 2.0 - Condition Orientated Regulatory Networks 2.0

Ricky Wai Tak Leung[1,2,^], Xinying Zhang[1,^], Zhuobin Chen[1,^], Yuyun Liang[1], Simei Huang[1], Zixin Yang[1], Xueqing Zong[1], Xiaosen Jiang[3], Runming Lin[4], Wenbin Deng[1,*], Yaohua Hu[5,*], Jing Qin[1,*]

[1] School of Pharmaceutical Sciences (Shenzhen), Shenzhen Campus of Sun Yat-sen University, Shenzhen, Guangdong 518107, China

[2] College of Professional and Continuing Education, The Hong Kong Polytechnic University, Kowloon, Hong Kong

[3] College of Life Sciences, University of Chinese Academy of Sciences, Beijing 100049, China.

[4] BGI-Shenzhen, Shenzhen, Guangdong 518103, China.

[5] Shenzhen Key Laboratory of Advanced Machine Learning and Applications, College of Mathematics and Statistics, Shenzhen University, Shenzhen, Guangdong 518060, China.

^Equal contribution. *Corresponding authors.

Table of contents

Figure/Table	Page number
Figure S1. Comparisons between CORN 2.0 (cyan) and CORN 1.0 (red) in number of enriched DO disease categories, enriched KEGG pathways and TFs.	3
Figure S2. Single-cell transcriptome atlas of lung adenocarcinoma.	4
Table S1. Summary of CORN update.	In a separate excel file
Table S2. The TRSNs matched with patients of lung adenocarcinoma.	In a separate excel file
Table S3. The TRSNs matched with DEG across different cell states associated with cell reprogramming.	In a separate excel file
Table S4. Suppelementary table for Figure 4.	In a separate excel file
Table S5 Suppelementary table for Figure 5.	In a separate excel file

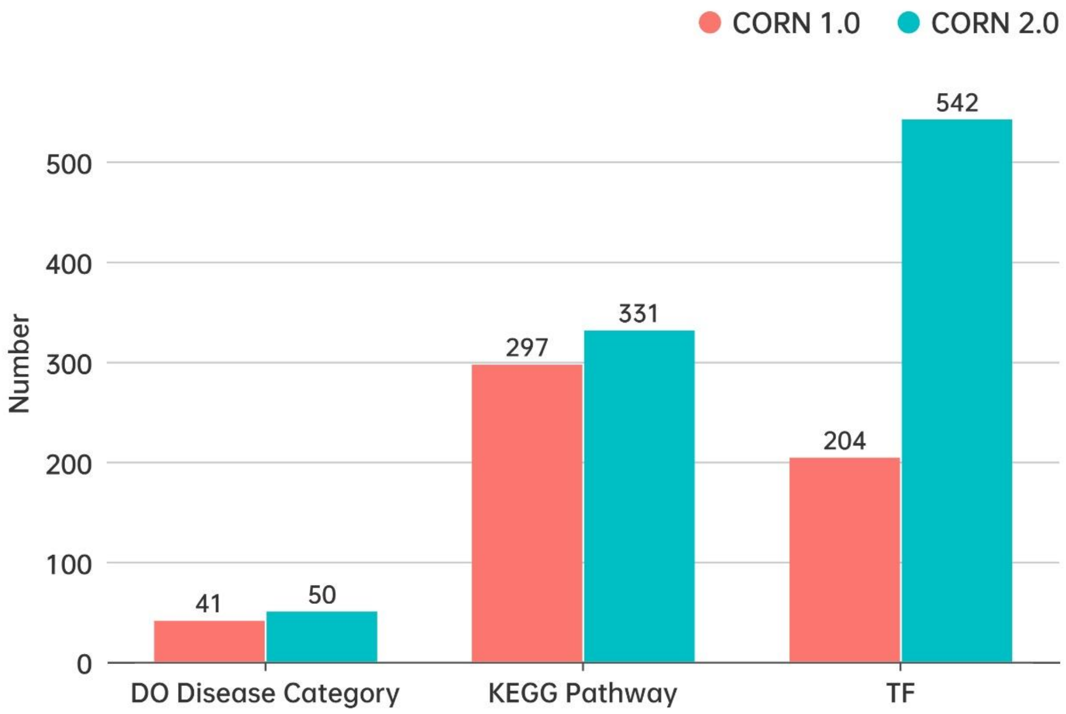

Figure S1. Comparisons between CORN 2.0 (cyan) and CORN 1.0 (red) in number of enriched DO disease categories, enriched KEGG pathways and TFs.

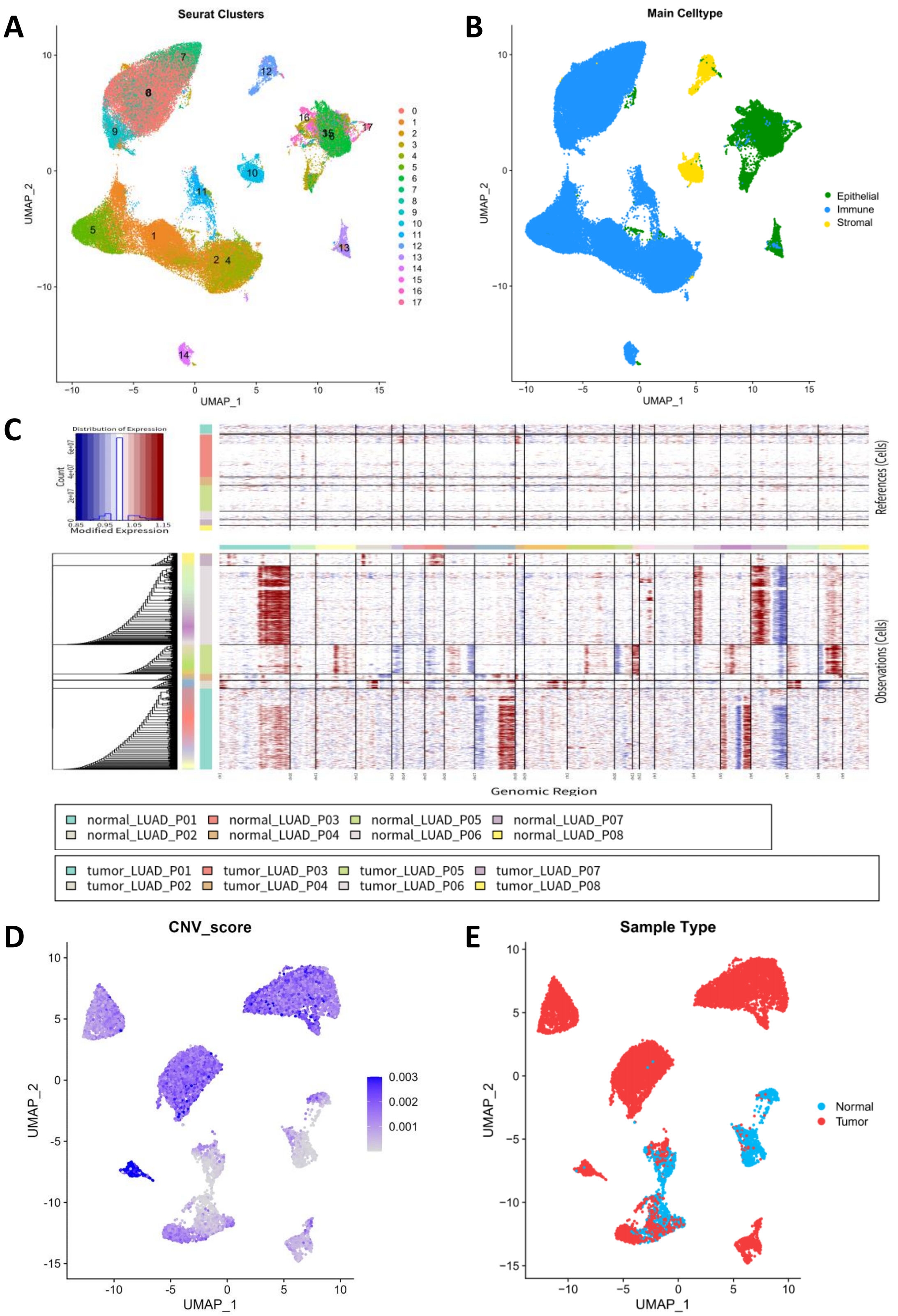

Figure S2. Single-cell transcriptome atlas of lung adenocarcinoma. UMAPs color-coded by (A) clusters and (B) main cell types. (C) Heatmap of CNVs from different patient sources. UMAPs color-coded by (D) CNV scores and (E) samples. See main text for details.
